# Supplementary material for: Making Neglect Invisible: A Qualitative Study among Nursing Home Staff in Norway
Source: Healthcare (Basel). 2023 May 12;11(10):1415. doi: 10.3390/healthcare11101415 (PMC10218649; doi:10.3390/healthcare11101415)
Supplement: Supplementary file 1 [file healthcare-11-01415-s001.zip › healthcare-2288533-Additional file S1-Interview-guide IJERPH.pdf]

Interview-guide

Main question

How is neglect communicated and reported among nursing home staff?

Introduce case studies or questions from survey-instrument if delayed response or difficulties to understand the topic.

Follow-up questions

- What is your response when you discover neglect?
- How do you follow-up when you discover neglect?
- What happens or can happen if you report this? What if you do not report?
- What prevents you from reporting neglect?
- What facilitates reporting neglect at your workplace?
- What written rules/procedures are there for reporting neglect at your workplace?
- What is decisive for whether you report neglect or not?
